# Biogenic fabrication and characterization of silver nanoparticles using aqueous-ethanolic extract of lichen (*Usnea longissima*) and their antimicrobial activity

**DOI:** 10.1186/s40824-018-0135-9

**Published:** 2018-09-21

**Authors:** Khwaja Salahuddin Siddiqi, M. Rashid, A. Rahman, Tajuddin, Azamal Husen, Sumbul Rehman

**Affiliations:** 1 Department of Chemistry, Aligarh Muslim University, Aligarh, Uttar Pradesh 202002 India; 2 Department of Saidla, Aligarh Muslim University, Aligarh, Uttar Pradesh 202002 India; 3 Department of Biology, College of Natural and Computational Sciences, University of Gondar, P.O. Box #196, Gondar, Ethiopia; 4 Department of Ilmul Advia (Unani Pharmacy), Aligarh Muslim University, Aligarh, Uttar Pradesh 202002 India

**Keywords:** Biosynthesis, *Usnea longissima*, Silver nanoparticles, Electron microscopy, Antimicrobial activity

## Abstract

**Background:**

Biogenic fabrication of silver nanoparticles from naturally occurring biomaterials provides an alternative, eco-friendly and cost-effective means of obtaining nanoparticles. It is a favourite pursuit of all scientists and has gained popularity because it prevents the environment from pollution. Our main objective to take up this project is to fabricate silver nanoparticles from lichen, *Usnea longissima* and explore their properties. In the present study, we report a benign method of biosynthesis of silver nanoparticles from aqueous-ethanolic extract of *Usnea longissima* and their characterization by ultraviolet–visible (UV-vis), Fourier transform infrared (FTIR) spectroscopy, transmission electron microscopy (TEM) and scanning electron microscopy (SEM) analyses. Silver nanoparticles thus obtained were tested for antimicrobial activity against gram positive bacteria and gram negative bacteria*.*

**Results:**

Formation of silver nanoparticles was confirmed by the appearance of an absorption band at 400 nm in the UV-vis spectrum of the colloidal solution containing both the nanoparticles and *U. longissima* extract. Poly(ethylene glycol) coated silver nanoparticles showed additional absorption peaks at 424 and 450 nm. FTIR spectrum showed the involvement of amines, usnic acids, phenols, aldehydes and ketones in the reduction of silver ions to silver nanoparticles. Morphological studies showed three types of nanoparticles with an abundance of spherical shaped silver nanoparticles of 9.40–11.23 nm. Their average hydrodynamic diameter is 437.1 nm. Results of in vitro antibacterial activity of silver nanoparticles against *Staphylococcus aureus*, *Streptococcus mutans, Streptococcus pyrogenes, Streptococcus viridans, Corynebacterium xerosis, Corynebacterium diphtheriae* (gram positive bacteria) and *Escherichia coli*, *Klebsiella pneuomoniae* and *Pseudomonas aeruginosa* (gram negative bacteria) showed that it was effective against tested bacterial strains. However, *S. mutans, C. diphtheriae* and *P. aeruginosa* were resistant to silver nanoparticles.

**Conclusion:**

Lichens are rarely exploited for the fabrication of silver nanoparticles. In the present work the lichen acts as reducing as well as capping agent. They can therefore, be used to synthesize metal nanoparticles and their size may be controlled by monitoring the concentration of extract and metal ions. Since they are antibacterial they may be used for the treatment of bacterial infections in man and animal. They can also be used in purification of water, in soaps and medicine. Their sustained release may be achieved by coating them with a suitable polymer. Silver nanoparticles fabricated from edible *U. longissima* are free from toxic chemicals and therefore they can be safely used in medicine and medical devices. These silver nanoparticles were stable for weeks therefore they can be stored for longer duration of time without decomposition.

## Background

Metal nanoparticles (NPs) have attracted much attention during recent years owing to their unique properties which are different from bulk material. These particles gained importance during recent years owing to their broad-spectrum application in a number of processes such as agriculture, cosmetics, healthcare, drug or gene delivery, medical devices, biosensor and catalysis [1–9] besides their antimicrobial properties [2, 10, 11]. Many metal NPs are essential nutrients to living system while some are toxic [12]. Their efficiency depends on their shape and size. Among the coinage metals silver has highest thermal and electrical conductivity. They may have multidimensional structure such as nanotubes and nanowires. A variety of methods for the fabrication of NPs have been developed but reduction reaction, photochemical reaction, thermal decomposition, electrochemical, sono-chemical and microwave assisted methods are prevalent these days. Although, these synthetic procedures are effective and high yielding they require chemicals which are often toxic and pollute the environment. However, these methods are not economical and sometime require expensive and hazardous chemicals which are difficult to handle. Green method of NP synthesis using plant extracts, bacteria, actinomycetes, fungi and enzymes are therefore, frequently used because of their environment friendly nature and bio compatibility [2, 13–16]. Major compounds found in plant extracts are generally glycosides, alkaloids, phenols, quinines, amines and terpenoids which convert silver ions to silver nanoparticles (Ag NPs) [2, 11]. Thus leaves, bark, flowers and seed extract of plants containing above chemicals are used as a source of reducing agents. For instance, Dhand et al. [17] have reported green synthesis of Ag NPs from roasted *Coffea arabica* seed extract. They were found to be highly crystalline with spherical and ellipsoidal shape. Average particle size ranged between 10 and 150 nm. It was observed that the particle size increased with decreasing concentration of AgNO_3_ solution. They were also effective against *Escherichia coli* and *Streptococcus aureus*. It was noted that smaller Ag NPs were more effective than the larger ones. In another study biosynthesis, biocompatibility and antibacterial activity of *Adathoda vasica* extract mediated Ag NPs have been thoroughly studied [18]. They showed significant antibacterial activity against *Vibrio parahaemolyticus* but were non-toxic to *Artemia naupli*. Since *Vibrio parahaemolyticus* causes vibriosis in shrimps (early mortality syndrome) biosynthesized Ag NPs have been used to protect them from this disease [19]. *Vibrio* infection also causes high mortality in Siberian tooth carps, milk fish, abalone and shrimps [20–22]. Overuse of vaccines and antibiotics have made them resistant. Since Ag NPs are known antibacterial substance they have been green synthesized from plant material and used frequently to prevent bacterial infections which are resistance to trivial drugs [2, 11].

The lichen, *Usnea longissima* belonging to Usneaceae family grows as moss on trees in temperate climate. They are slowest growing plants living in symbiosis with algae, fungi and perennial trees. Different genera of lichens are used in curing dyspepsia, amenorrhea and vomiting. Lichens produce secondary metabolites which are used as crude drugs. It contains mainly usnic acid and its derivatives called usenamines, usone and iso-usone [23]. Three compounds containing OH and NH_2_ groups have been shown to inhibit the growth of human hepatoma, HepG2 cells with significant IC50 values between 6.0–53.3 μM. This value is lower than that found for methotrexate (IC50 value of 15.8 μM) under the same condition. *U. longissima* exhibits myriad biological properties such as antitumor, antiviral, antimicrobial, anti-inflammatory and insecticidal activities. Since it is known to damage the liver, its application in human system is limited [24–26] even though it is used to treat ascariasis [27] and fractured bones. Its extract is known to contain monosubstituted phenyls, depsides, anthraquinones, dibenzofurans and terpenoides which have been shown to exhibit insecticidal and antioxidant activities [23, 28–30]. A number of bacteria and fungi (*E. coli*, *Candida albicans*, *Bacillus subtilis*, *Mycobacteriun smegmatis*, *Trichophyton rubrum* and *Aspergillus niger*) have been used to investigate the in vitro activity of usnic acid derivatives [23, 31, 32]. Usnic acid derivatives are cytotoxic and antimicrobial it is also used as an expectorant and in the treatment of ulcer. It has been shown by Nishitoba et al. [28] that all depsides and orcinol derivatives of *U. longissima* act as growth inhibitor of lettuce seedlings. Inhibition of tumour promoter induced Epstein-Barr virus by *U. longissima* extract has been shown to exhibit highest inhibition activity [33]. Methanol extract of *U. longissima* has also exhibited antioxidant activity [34]. Antiulcerogenic effect of *U. longissima* water extract against indomethacin induced ulcer in rat has been investigated [29]. The extract showed moderate antioxidant activity when compared with trolox and ascorbic acid as positive antioxidant [29]. Biosorption of trace amounts of Au(III) and Cu(II) by *U. longissima* biomass has been investigated [35]. It is surprising that effective absorption of both the metals occurs either at pH 2 or pH 8 within 75 min. It has been found that 1 g of dry lichen absorbed 9.4 mg Au(III) and 24.0 mg Cu(II). The recovery of metals is nearly quantitative (> 90%).

Several compounds from *U. longissima* have been isolated and identified but no effort seems to have been made to synthesis Ag NPs. Although, Ag NPs alone have numerous qualities, bio-functionalized NPs are more effective against pathogenic microbes such as bacteria, virus and fungi. Biosynthesized Ag NPs using edible *U. longissima* is free from toxic chemicals and hence they can be safely used in medicine and medical devices. We are, therefore, reporting, for the first time, the biosynthesis of Ag NPs from *U. longissima* in 50:50 water- ethanol extract and their characterization by ultraviolet–visible (UV-vis), Fourier transform infrared (FTIR) spectroscopy, size distribution, transmission electron microscopy (TEM) and scanning electron microscopy (SEM) analyses. Their antibacterial activity against some clinical isolates of bacterial strains (six-gram positive and three-gram negative) has also been investigated.

## Methods

### Chemicals, plant material and instrumentation

**Fig. 1:**
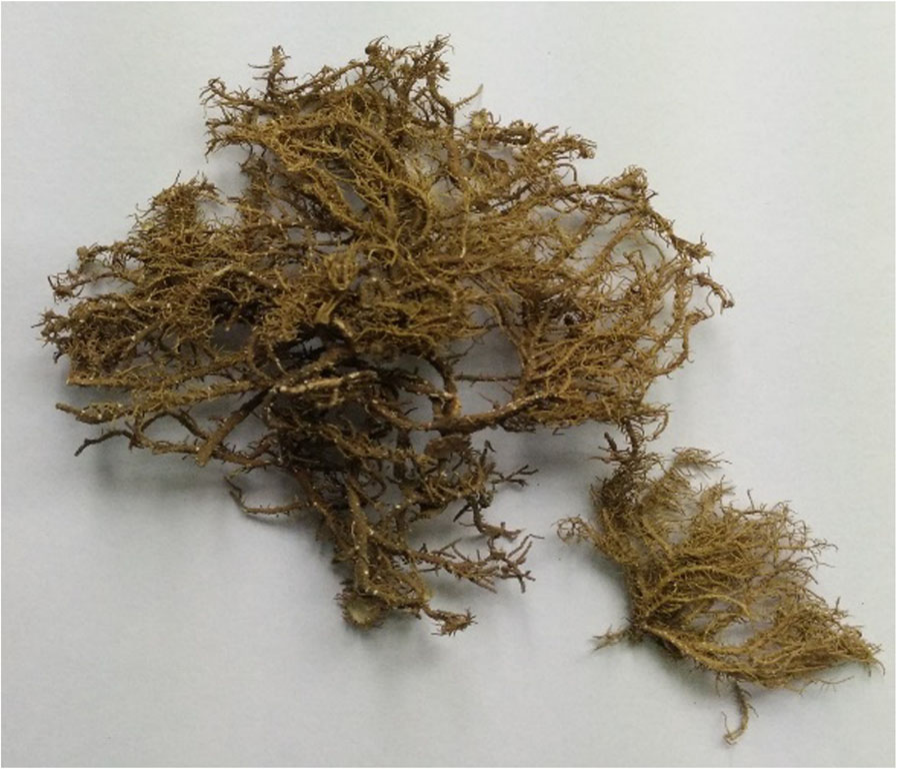
*Usnea longissima*

AgNO_3_ (Merck, India Ltd.), ethanol (AR grade) and double distilled water were used. Aqueous solution of poly(ethylene glycol) (Merck, India Ltd.) was used. *Usnea longissima* was procured from the pharmacy unit of Aligarh Muslim University, Aligarh, India (Fig. 1). UV-vis spectral measurements were done with an Elico Spectrophotometer between 200 and 500 nm. Size distribution was determined by Malvern Instruments Ltd., Zetasizer Ver. 7.11. FTIR spectra were recorded with Perkin-Elmer Spectrometer, FTIR spectrum ONE, in 4000–400 cm^− 1^ region as KBr disc. TEM Images of Ag NPs were obtained using JEOL, JEM 2100 transmission electron microscope at 190 KV. Samples were prepared using a drop of colloidal solution of Ag NPs on a carbon coated copper grid and allowing the above sample to completely dry in a vacuum desiccator. The sediment particles obtained were used for scanning FTIR spectra. SEM images were obtained with JEOL, JSM 6510LV scanning electron microscope.

### Synthesis of ag NPs

Ag NPs were prepared from aqueous-ethanolic extract of *U. longissima*. Lichen was gently washed with distilled water to remove dust. It was subsequently dried at 60^0^ C and powdered. Ten g of this dry powder was refluxed in 100 ml ethanol-distilled water (50:50) mixture for 3 h, cooled to room temperature and centrifuged at 10,000 rpm to remove the solid mass. Ten ml of this extract at pH 7 was taken in an Erlenmeyer flask and 1 ml of 0.01 M solution of AgNO_3_ was added to start the reduction of silver ions to Ag NPs. The mixture was vigorously stirred on a magnetic stirrer for ten to fifteen min and incubated in dark to protect the contents from sunlight. Colour change was regularly monitored. Reaction was completed only after 72 h showing purple colour. Reaction mixture was then centrifuged at 10,000 rpm to separate NPs from the liquid. It was decanted and the supernatant was further centrifuged to isolate any NP left in the solvent. The sample thus obtained was stable for weeks although the yield was very low (35%). All manipulations were done at ambient temperature.

### Evaluation of antibacterial activity

Ag NPs thus obtained were tested for antimicrobial activity against *Staphylococcus aureus*, *Streptococcus mutans, Streptococcus pyrogenes, Streptococcus viridans, Corynebacterium diphtheriae* and *Corynebacterium xerosis* (six-gram positive bacteria) and *Escherichia coli*, *Klebsiella pneuomoniae* and *Pseudomonas aeruginosa* (three-gram negative bacteria) obtained from Department of Microbiology, Jawaharlal Nehru Medical College & Hospital, Aligarh Muslim University, Aligarh, India. The solid media namely Nutrient Agar No.2 (NA) (M 1269S-500G, Himedia Labs Pvt. Ltd., Bombay, India) was used for preparing nutrient plates, while Nutrient Broth (NB) (M002-500G, Himedia Labs Pvt. Ltd., Bombay, India) was used for the liquid culture media. Antibacterial activity was evaluated by agar well diffusion method. All the microbial cultures were adjusted to 0.5 McFarland standards, which is visually comparable to a microbial suspension of 1.5 Х 10^8^ cfu/ml. Agar medium (20 ml) was poured into each petri plate and were swabbed with a colony from the inoculums of the test microorganisms and kept for 15 min for adsorption. Using sterile cork borer of 6 mm diameter wells were bored into the seeded agar plates. They were loaded with 100 μl of dimethylsulphoxide (DMSO) of 2 mg/ml. All the plates were incubated at 37 °C for 24 h. Antimicrobial activity was evaluated by measuring the zone of growth inhibition against the tested gram positive and gram negative bacteria with Antibiotic Zone Scale (PW297, Himedia Labs Pvt. Ltd., Mumbai, India), which was held over the back of the inverted plate. It was held a few inches above a black, non-reflecting background and illuminated with reflected light. The medium with DMSO as solvent was used as a negative control whereas media with Ciprofloxacin (5 μg/disk as standard antibiotic for gram positive) and Gentamicin (10 μg/disk as standard antibiotic for gram negative) were used as positive control. The experiments were performed in triplicates.

## Results and discussion

### UV-vis spectra

It is known that when Ag NPs are formed, colour of the solution containing both the NPs and plant extract turns dark brown or purple depending on the presence of organic molecules in the extract. In our case, AgNO_3_ was mixed with aqueous- ethanolic extract of *U. longissima* and incubated at room temperature; its colour turned purple after 72 h. It did not show any significant change thereafter which confirms the formation of Ag NPs according to the following equation.

$$ {\mathrm{Ag}\mathrm{NO}}_3+{\mathrm{NR}}_3={\mathrm{Ag}}^0+\mathrm{NR}{3}^{+}+{\mathrm{H}}^{+}+{{\mathrm{NO}}_3}^{-} $$

**Fig. 2:**
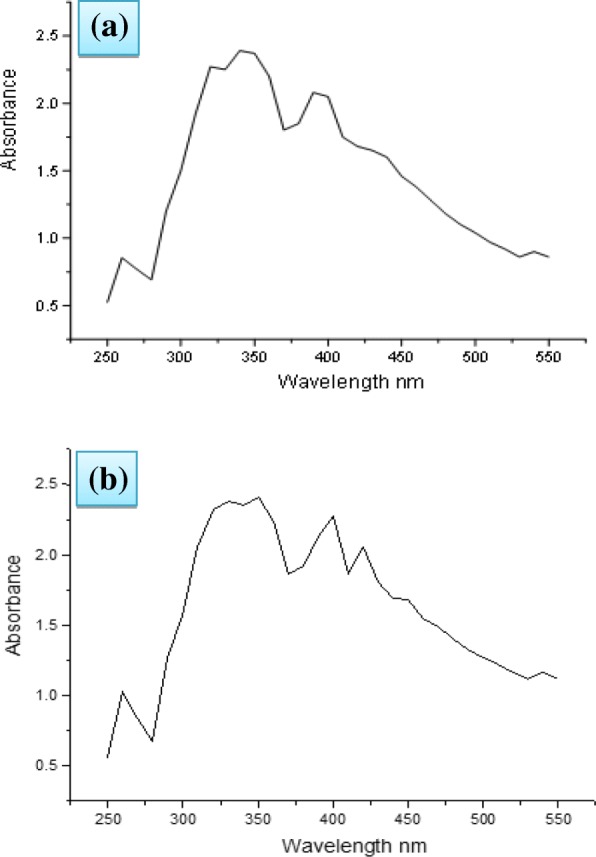
UV-vis spectrum of (**a**) silver nanoparticles and (**b**) poly(ethylene glycol) coated silver nanoparticles at 25 °C

**Fig. 3:**
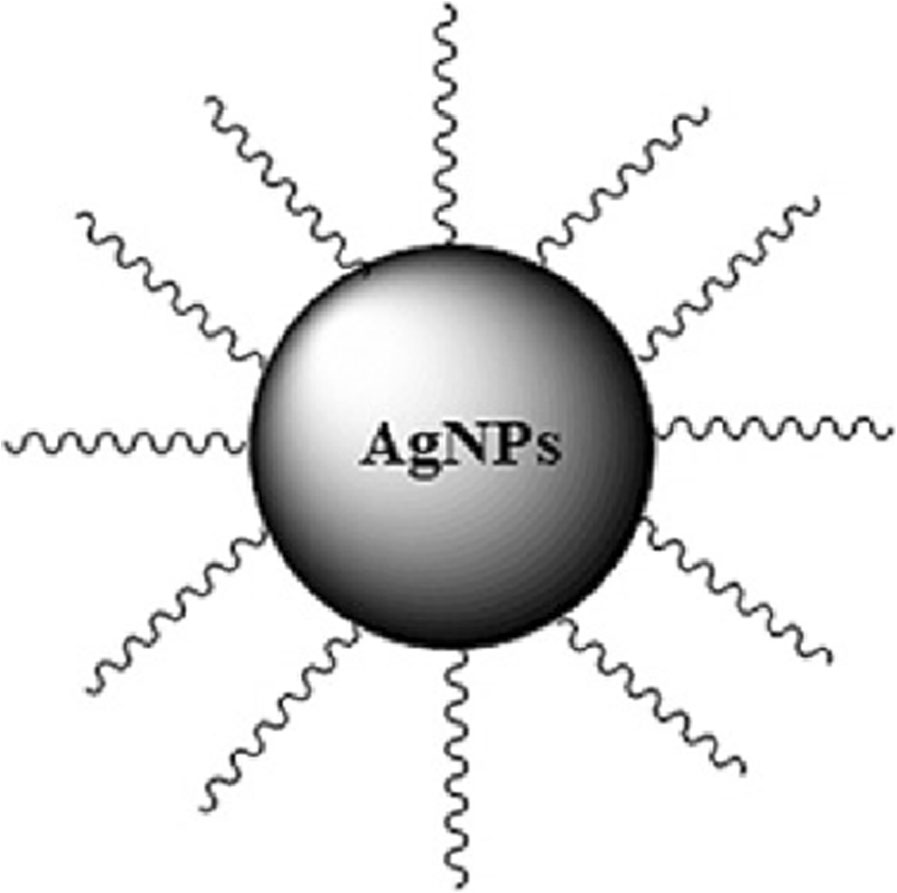
Poly(ethylene glycol) coated silver nanoparticles

The UV-vis spectrum of this colloidal solution was run from 200 to 500 nm at room temperature which displayed peaks at 350 and 400 nm. Highest peak at 400 nm has been attributed to the excitation of surface plasmon resonance (SPR) of Ag NPs (Fig. 2a). Photo-oxidation of chemical constituents present in the extract may also have occurred [36]. Profile of the UV-vis spectrum depends on the concentration of substrate and silver ions. However, when aqueous solution of poly(ethylene glycol) – PEG, was added to the above solution, new peaks at 424 and 450 nm were observed (Fig. 2b). It is probably due to coating of Ag NPs with the polymer as shown in figure (Fig. 3). Colour of these NPs remained unchanged even after several weeks [37]. It has been observed that when the colloidal solution containing Ag NP is slowly heated up to 60 °C the colour intensity increases with increasing temperature and NPs are quickly formed. It demonstrates the effect of temperature on the biosynthesis of Ag NPs. Absorption peaks in the UV-vis spectrum are related to the shape of Ag NPs. According to the criterion of Zhang and Nogues [38] the peaks at 385, 435, 465 and 515 nm correspond to cubical Ag NPs, those at 462 for truncated cubes, at 430 cuboctahedral and 400 nm peak for spherical NPs. Since we have observed major peak at 400 nm Ag NPs are supposed to be mainly spherical though the presence of small amount of other types of NPs cannot be ignored.

**Fig. 4:**
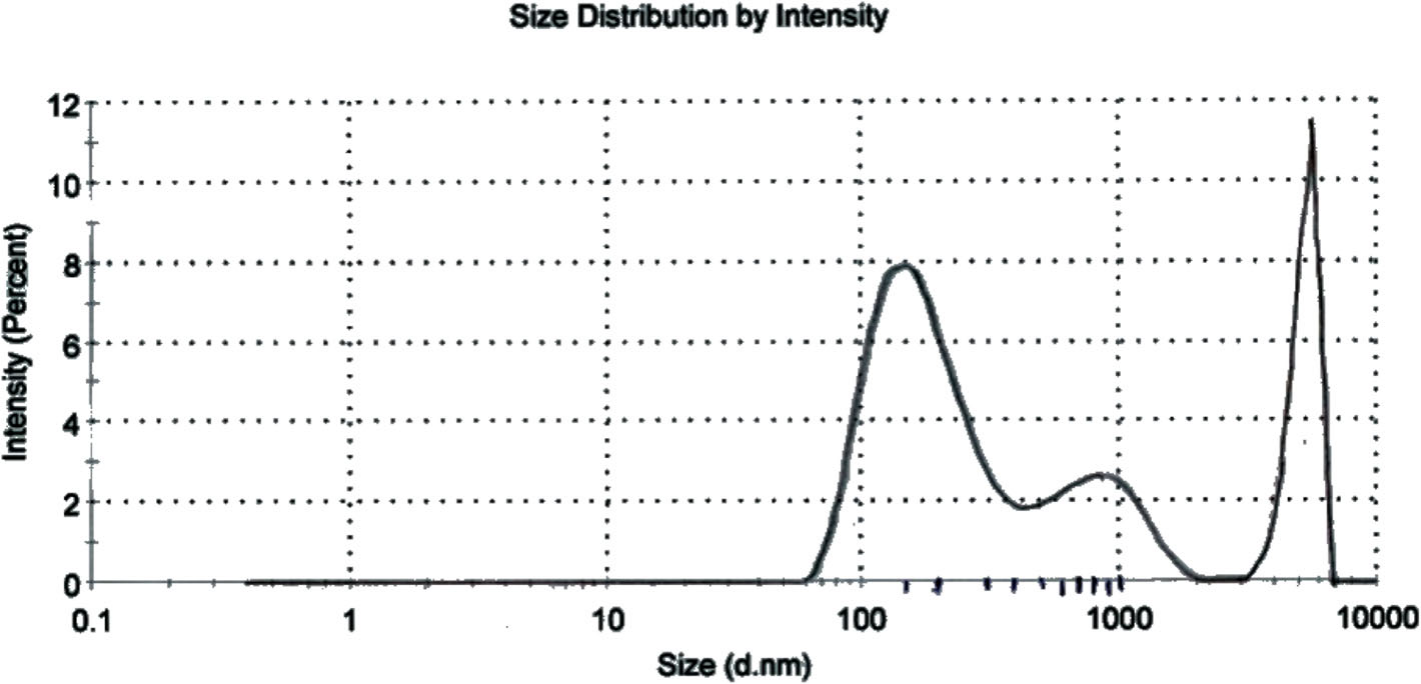
Particle size distribution of *Usnea longissima* mediated silver nanoparticles

Size distribution was performed using water as dispersant at a count rate of 271.6 k cps. There are three types of Ag NPs present in the colloidal solution. Their hydrodynamic diameters are very large as shown in Fig. 4. However, the peak-1 shows the abundance of particles with average hydrodynamic diameter of 184.5 nm with an intensity of 59.4% but overall average is 437.1 nm. This may be due to aggregation of the particles in the solvent. The hyddrated NPs are always larger than the isolated ones because of the aggregation of water molecules around them. Since the surface area of aggregated NPs is decreased they would not be in direct contact with microbes and their antibacterial efficiency will obviously decrease.

### TEM and SEM

**Fig. 5:**
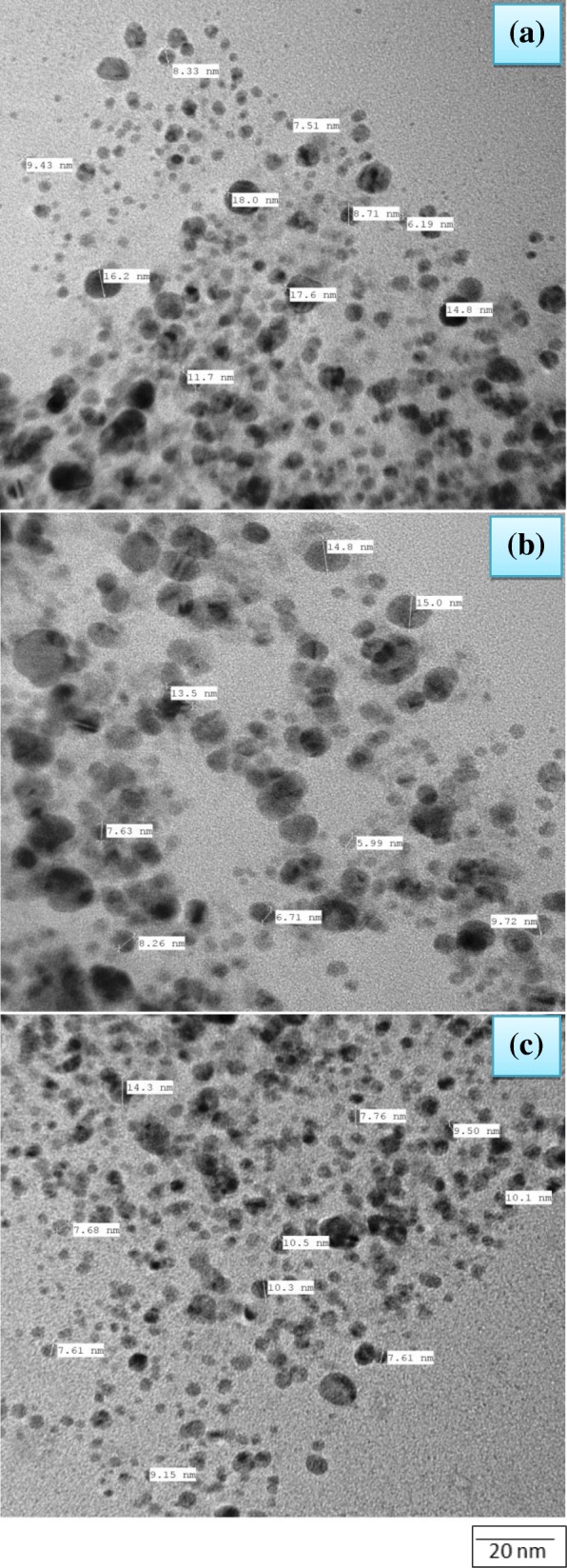
TEM images of silver nanoparticles; (**a**) under 80,000 magnification (average size, 11.83 nm), (**b**) under 100,000 magnification (average size, 10.20 nm) and (**c**) under 80,000 magnification (average size, 9.44 nm)

TEM images (Fig. 5) showed that Ag NPs in our case are mainly spherical in shape. There is very narrow range in the size of NPs. Average particle size varies between 9.4 and 11.83 nm. TEM images show spherical morphology with an average size of 10.49 nm (Fig. 5a-c). It has also been observed that these are much smaller in size than those recently reported [39].

**Fig. 6:**
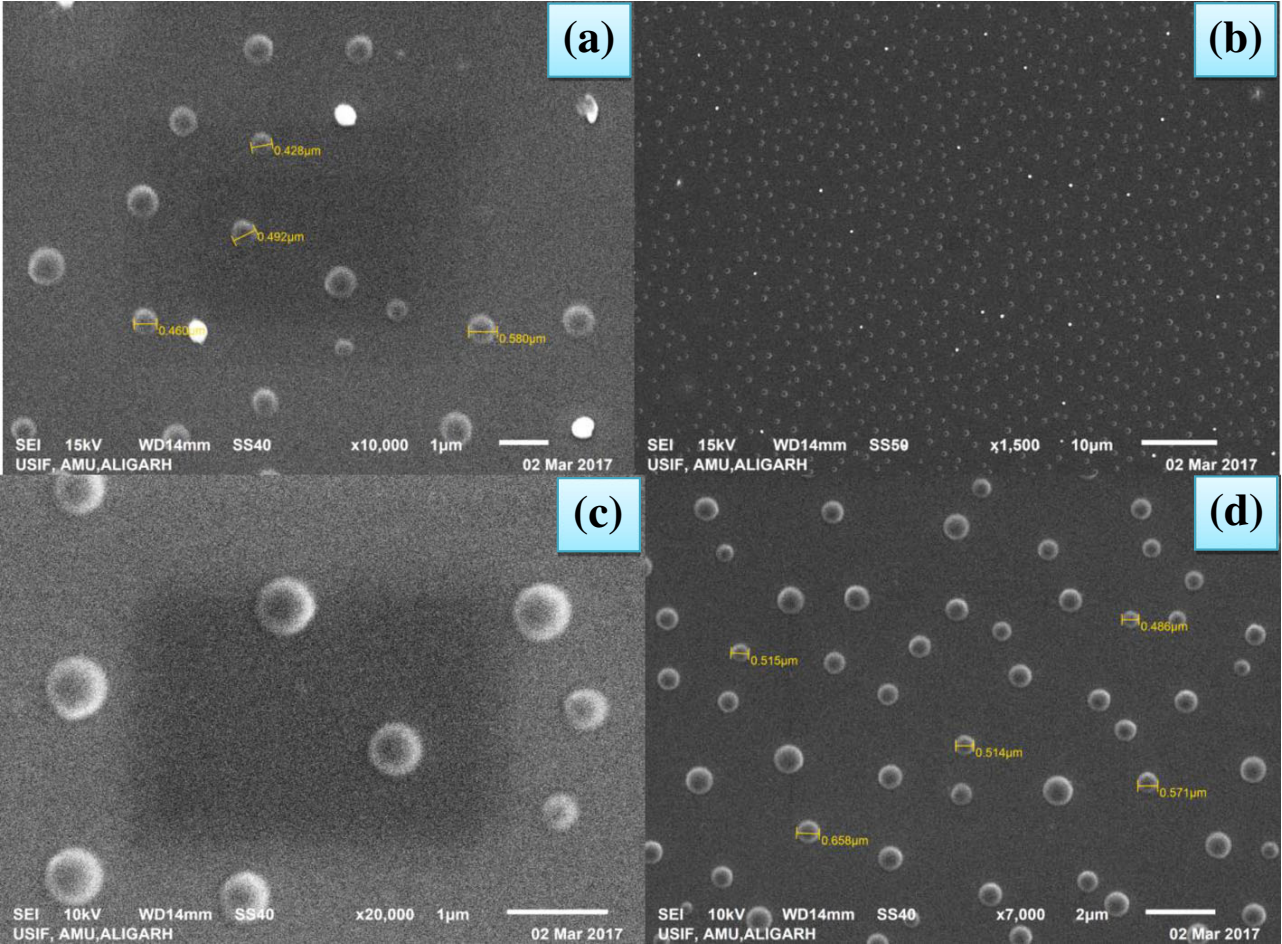
SEM images of silver nanoparticles; (**a**) under 10,000 magnification, (**b**) under 1500 magnification, (**c**) under 20,000 magnification and (**d**) under 7000 magnification.

The topology and size were also confirmed by SEM images (Fig. 6a-d) showing the presence of small and uniformly spherical shaped Ag NPs with smooth surface and very narrow distribution range of 9.4–11.83 nm [40]. The larger particles are formed due to aggregation of Ag NPs otherwise they appear to be segregated. The cluster must have been formed due to evaporation of the solvent during sample preparation. The scattered shiny dots appearing in the SEM images are due to uncoated free Ag NPs which look like shining stars in milkyway (Fig. 6b) in dark night [41].

### IR spectrum

**Fig. 7:**
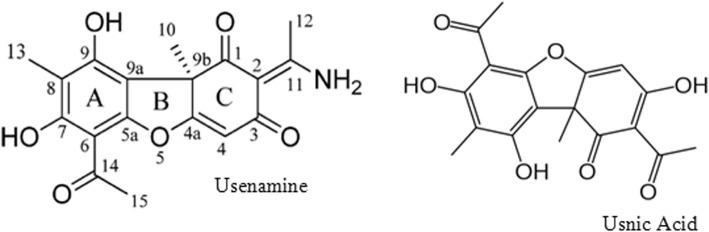
Structures of usenamine and usnic acid

**Fig. 8:**
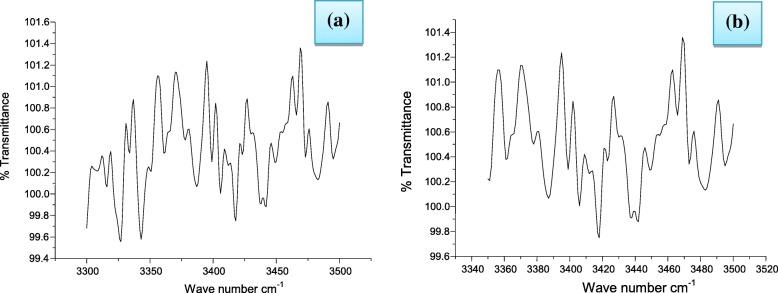
FTIR spectra of (**a**) aqueous-alcoholic extract and *(***b***)* silver nanoparticles

FTIR spectrum was run to identify the involvement of biomolecules present in *U. longissima* extract for the reduction of AgNO_3_ to Ag NPs. It is known to contain phenol, amines, aldehydes and ketones besides many other compounds in traces. However, usnic acid and usenamine (Fig. 7) are dominant compounds in aqueous- ethanolic extract of *U. longissima* which interact with AgNO_3._ Since all these compounds are excellent reducing agents, they undergo changes in stretching frequencies of their functional groups as a consequence of reduction of AgNO_3_ to Ag NPs. IR spectrum (Fig. 8) is very complicated because of the overlap of frequencies in the same region. However, we have attempted to identify the shifts in stretching vibrations after the formation of Ag NPs. Primary amines exhibit two N-H stretching frequencies in 3500–3300 cm^− 1^ region which have been found to appear at 3400 and 3455 cm^− 1^ in the NPs containing *U. longissima extract* (Fig. 8). The band in 1600–1500 cm^− 1^are due to CO stretching but amide bands also appear in the same region of spectrum. We have observed amide I and amide II bands at 1650 and 1540 cm^− 1^. A band at 1560 cm^− 1^ has been assigned to (C=O) stretching frequency. The COO^−^ group generally appears above 1600 cm^− 1^ but overlaps with amide II band [42]. These spectral results indicate the involvement of organic molecules in the reduction of AgNO_3_ leading to the formation of Ag NPs.

### Antibacterial screening

Results of in vitro antibacterial activity of Ag NPs against *Staphylococcus aureus*, *Streptococcus mutans, Streptococcus pyrogenes, Streptococcus viridans, Corynebacterium diphtheriae* and *Corynebacterium xerosis* (gram positive bacteria) and *Escherichia coli*, *Klebsiella pneuomoniae* and *Pseudomonas aeruginosa* (gram negative bacteria) are presented in Table 1. The zone of inhibition suggests that Ag NPs are weekly toxic to both gram positive and gram negative bacteria. Ag NPs with larger surface area provide a better contact with microorganisms [2, 6, 11]. Thus, these particles may penetrate the bacterial cell membrane or attach to the bacterial surface and inhibit their replication [43, 44]*.* In our experiment, Ag NPs have been found to be most effective against *E. coli*. It has been reported that antibacterial efficiency is increased by lowering the particle size [45]. Usually NPs attach on the cell wall of bacteria and damage membrane and respiration system leading to cell death [11, 43]. Toxicity of smaller NPs was greater than those of larger ones because the smaller ones can easily adhere to bacterial cell wall [11, 46].

**Table 1:** Mean zone of inhibition (in mm)

Bacterial strains	Silver nanoparticles	Negative control*	Positive control**
*Staphylococcus aureus*	10.6 ± 0.50^a^	6.2 ± 0.20	28.0 ± 0.89
*Streptococcus mutans*	6.5 ± 0.89^a^	5.8 ± 0.40	29.2 ± 0.83
*Streptococcus pyrogenes*	15.6 ± 0.40^a^	6.2 ± 0.20	29.2 ± 0.48
*Streptococcus viridans*	14.6 ± 0.24^a^	6.2 ± 0.20	25.6 ± 0.20
*Corynebacterium xerosis*	13.6 ± 0.50^a^	6.2 ± 0.20	22.8 ± 0.58
*Corynebacterium diphtheriae*	6.2 ± 0.37^a^	6.2 ± 0.20	22.2 ± 0.48
*Escherichia coli*	20.8 ± 0.02^a^	6.2 ± 0.20	26.8 ± 0.48
*Klebsiella pneuomoniae*	16 ± 0.31^a^	6.2 ± 0.20	26.4 ± 0.24
*Pseudomonas aeruginosa*	7 ± 0.31^a^	6.2 ± 0.20	21.8 ± 0.37

Values are expressed as mean ± SD (*n* = 3) and valued followed by same letter are not significantly different at the *p* < 0.0001as determined by Duncan’s Multiple Range Test; * indicates dimethyl sulphoxide, ** indicates standard drug (Ciprofloxacin for gram positive and Gentamicin for gram negative bacterial strains)

### Mechanism of action

Silver ions penetrate into cytoplasm; denature the ribosome leading to the suppression of enzymes and proteins which eventually arrest their metabolic function resulting in apoptosis of bacteria. Bactericidal activity is due to silver ions released from Ag NPs as a consequence of their interaction with microbes [11]. However, four possible mechanisms of antibacterial activity of Ag NPs have been proposed (i) interference during cell wall synthesis (ii) suppression during protein biosynthesis (iii) disruption of transcription process and (iv) disruption of primary metabolic pathways [17]. Each mechanism involves structural changes, biochemical changes and charges on both the silver ions and bio molecules in the microbial cells. Ag NPs also inhibit the proliferation of cancer cell lines by different modes of action [47]. They mediate and amplify the death signal by triggering the activation of Caspase-3 molecule. The DNA splits into fragments by Caspase-3. Ag NPs may interfere with the proper functioning of cellular proteins and induce subsequent changes in cellular chemistry. Sometimes Ag NPs alter the function of mitochondria by inhibiting the catalytic activity of lactate dehydrogenase. Ag NPs may also cause proliferation of cancer cells by generating ROS which ultimately leads to DNA damage.

## Conclusion

Few species of lichens have rarely been exploited in the production of NPs; in the present investigation we successfully fabricated Ag NPs by bio-reduction of silver nitrate from aqueous-alcoholic extract of *U. longissima* at room temperature. Size distribution shows the presence of three types of Ag NPs, the average diameter of which is 437.1 nm. However, NPs with hydrodynamic diameter of 184.5 nm are in abundance. It has been observed from SEM images that NPs are mainly spherical in shape. There is not very large variation in their size (9.4–11.3 nm). Ag NPs are antibacterial and their sustained release may be achieved by coating them with a suitable polymer. They are highly effective against *E. coli* and *K. pneuomoniae*, although *S. mutans*, *C. diphtheriae* and *P. aeruginosa* are resistant to it. In the present work, the lichen acts as reducing as well as capping agent. These Ag NPs were stable for weeks therefore they can be stored for longer duration of time. However, their slow oxidation to silver ions cannot be prevented.
